# Experimental visualization of sneezing and efficacy of face masks and shields

**DOI:** 10.1063/5.0030101

**Published:** 2020-11-01

**Authors:** Venugopal Arumuru, Jangyadatta Pasa, Sidhartha Sankar Samantaray

**Affiliations:** Applied Fluids Group, School of Mechanical Sciences, Indian Institute of Technology Bhubaneswar, Bhubaneswar 752050, India

## Abstract

In the present work, we propose and demonstrate a simple experimental visualization to simulate sneezing by maintaining dynamic similarity to actual sneezing. A pulsed jet with Reynolds number Re = 30 000 is created using compressed air and a solenoid valve. Tracer particles are introduced in the flow to capture the emulated turbulent jet formed due to a sneeze. The visualization is accomplished using a camera and laser illumination. It is observed that a typical sneeze can travel up to 25 ft in ∼22 s in a quiescent environment. This highlights that the present widely accepted safe distance of 6 ft is highly underestimated, especially under the act of a sneeze. Our study demonstrates that a three-layer homemade mask is just adequate to impede the penetration of fine-sized particles, which may cause the spreading of the infectious pathogen responsible for COVID-19. However, a surgical mask cannot block the sneeze, and the sneeze particle can travel up to 2.5 ft. We strongly recommend using at least a three-layer homemade mask with a social distancing of 6 ft to combat the transmission of COVID-19 virus. In offices, we recommend the use of face masks and shields to prevent the spreading of droplets carrying the infectious pathogen. Interestingly, an N-95 mask blocks the sneeze in the forward direction; however, the leakage from the sides and top spreads the sneeze in the backward direction up to 2 ft. We strongly recommend using the elbow or hands to prevent droplet leakage even after wearing a mask during sneezing and coughing.

## INTRODUCTION

With the outbreak of the new pandemic “COVID-19,” humanity is struggling to combat and recover from social-economical losses. Over the globe, scientists and medical experts are engaged in developing a precise understanding of the transmission of COVID-19. The spreading of the infectious pathogen responsible for COVID-19 is mainly through droplets ejected during coughing and sneezing (Jones and Brosseau, 2015; Asadi *et al.*, 2020; and Bourouiba, 2020). The multiphase turbulent cloud formed during coughing and sneezing consists of hot and moist air and suspended droplets (Scharfman *et al.*, 2016). The larger droplet follows a ballistic projectile, and under the influence of gravity and aerodynamic drag, it decelerates and travels considerably a smaller distance before landing on surfaces (Tellier, 2006; Wells, 1934). However, the smaller diameter droplets and particles (<5 *µ*m–10 *µ*m) follow the turbulent gas cloud and travel a considerable distance based on the strength of the sneeze or cough, background mean flow, and turbulence (Bourouiba *et al.*, 2014; Bourouiba, 2020). These small aerosolized particles may contain the infectious pathogen, which may be directly inhaled or may remain suspended in the air for long time and may cause airborne transmission of infection. Parameters such as the size of the droplets, injection angle of the micro-droplets, cloud opening angle, velocity, and atmospheric conditions hugely affect the spreading of saliva (Pendar and Páscoa, 2020). The saliva droplets from a human cough carrying the virus can travel 2 m when the wind speed is zero. With the variation of wind speed in the range of 4 km/h–15 km/h, droplets can race to 6 m (Dbouk and Drikakis, 2020a; 2020b). The chances of virus survival inside the droplet are explored by Bhardwaj and Agrawal (2020a; 2020b). They observed a weak correlation between the drying time and the growth rate of the spread of COVID-19 in various cities. Furthermore, they proposed design guidelines for tailoring the surface wettability to combat the spread of infection of COVID-19 (Bhardwaj and Agrawal, 2020b). Li *et al.* (2020) and Wang *et al.* (2020) computationally simulated the possibility of virus transmission from the turbulent cloud generated during toilet and urinal flushing and recommended using face masks in public bathrooms. With the spread of “COVID-19,” countries enforce strict norms to wear masks and maintain social distancing. While masks have been found to reduce the risk of cross-infection from an infected to a healthy individual, social distancing ensures that the direct exposure to droplets is significantly reduced (MacIntyre *et al.*, 2009; MacIntyre and Chughtai, 2020). The efficacy of standard masks in preventing droplet transmission during breathing and coughing is well documented (Ha’Eri and Wiley, 1980; Johnson *et al.*, 2009; Lindsley *et al.*, 2012; 2014; Zayas *et al.*, 2013; Leung *et al.*, 2020; Zhou *et al.*, 2018; and Verma *et al.*, 2020a; 2020b). However, the penetration of small aerosolized particles or droplets (∼1 *µ*m to 10 *µ*m) through standard and non-standard masks during normal and severe sneezing is scarcely addressed. These small aerosolized droplets may cause airborne transmission of COVID-19 (Zhang *et al.*, 2020). Flow visualization had played a pivotal role in understanding the fluid dynamics of coughing and sneezing (Bourouiba *et al.*, 2014; Dudalski *et al.*, 2020; and Vadivukkarasan *et al.*, 2020). Flow visualization using the optical schlieren system was employed by Lewis *et al.* (1969) and Clark and Edholm (1958) to understand human thermal plume. Tang *et al.* (2009; 2011) reported schlieren visualization to understand the spreading of cough and showed the effectiveness of reducing the jet spread by a surgical or N-95 mask. Nishimura *et al.* (2013) employed high-speed imagining techniques to visualize the droplets emulated during coughing and sneezing. With recent acknowledgment from the World Health Organization (WHO) regarding the possibility of airborne COVID-19 virus transmission, it is even more crucial to study the behavior of smaller size droplets (∼5 *µ*m–10 *µ*m) in the turbulent cloud generated during coughing and sneezing (Mittal *et al.*, 2020; World Health Organisation, 2020).

Recently, Verma *et al.* (2020a; 2020b) reported an experimental study based on laser illumination flow visualization to simulate the propagation of fine aerosol particles using smoke during coughing. They proposed a few guidelines for social distancing based on the reach of the smoke analyzed from visualization. However, in their analysis, they have not reported the complete dynamic similarity considering the velocity and timing of actual coughing. Simha and Rao (2020), using schlieren visualization, reported that a typical cough might travel at least 1.5 m–3 m. A sneeze is not considered in both studies, which is very common and the most violent spasmodic expiration. Seasonal changes cause frequent sneezing, especially in people suffering from allergic rhinitis. A recent computational study by Busco *et al.* (2020) revealed that droplets of diameter ∼10 *µ*m may remain suspended in the air for more than 50 s during sneezing. A sneeze carrying smaller diameter droplets may travel a considerable distance through face masks. Hence, the present study’s objective is to document the reach of a typical sneeze in a quiescent environment and evaluate the efficacy of various standard and non-standard face masks and face shields under the influence of sneeze.

## EXPERIMENTAL SETUP

**FIG. 1.**
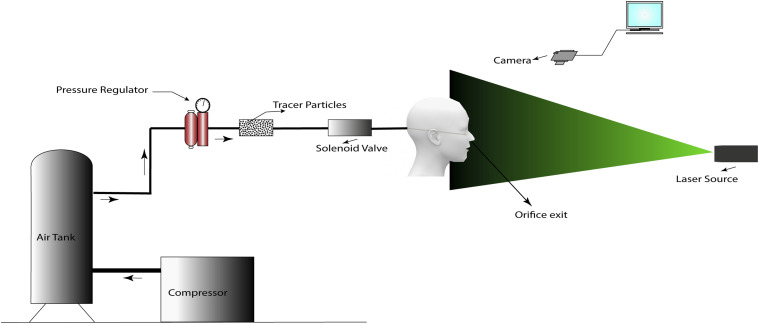
Experimental setup details.

**FIG. 2.**
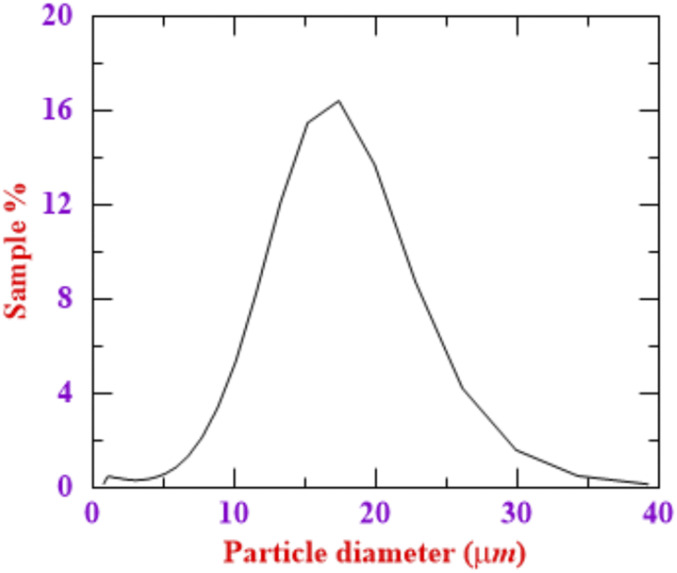
Particle size distribution of tracer particles.

Experiments are performed using a compressed air supply to simulate the flow after sneezing. A schematic diagram of the experimental setup is shown in Fig. 1. The jet issues out of a circular orifice of diameter 10 mm fitted with a solenoid valve. The nozzle exit area of ∼80 mm^2^ is similar to the average nostril area of humans (Zaidi *et al.*, 2017; Han *et al.*, 2013). The average volume inhaled by humans is about 500 ml. The sneezing velocity varies from 10 m/s to 50 m/s, and the duration of a sneeze for humans varies from 0.06 s to 0.3 s (Tang *et al.*, 2013). The solenoid valve is programmed to open for 0.2 s to simulate the act of sneezing, which establishes an average air velocity of 40 m/s at the nozzle exit. The motivation for selecting the velocity is based on the Reynolds number for sneezing reported by Bourouiba *et al.* (2014). In their study, they reported the Reynolds number for sneezing as Re = 40 000. We have selected a Reynolds number Re ∼30 000, and accordingly, we have adjusted the velocity at the nostril exit. During sneezing, the droplets are ejected from both nostrils and mouth; however, most of the volume is expelled through the nose with high velocity. Hence, we have considered only the nostril area. The velocity at the exit of the nose of a standard mannequin is measured using a hotwire anemometer. The mannequin nose’s height from the ground is 5. 6 ft and inclined at 25 ± 1° with the horizontal. The Reynolds number of the turbulent jet is ∼30 000. A pressure regulator is connected to the inlet of the solenoid valve to set the inflow pressure. Corn starch as a tracer particle is inserted into the pipeline just upstream of the solenoid valve for flow visualization. The mean particle size of the tracer particle obtained from HORIBA 950 LAV2 particle size analyzers is 14 *µ*m, measured with an accuracy of 0.6%. The particle size distribution is shown in Fig. 2. The mean droplet size distribution for sneezing reported in the literature varies in the range of 5 *µ*m–80 *µ*m (Han *et al.*, 2013). Such a difference in the particle size is attributed to various reasons such as the influences of the measurement method, the limitation of the instrument, the evaporation effects of the droplets, and the biological dynamic mechanism and characteristic of sneeze (Han *et al.*, 2013; Xie *et al.*, 2007). Considering a droplet of mean diameter 5 *µ*m–10 *µ*m suspended in a turbulent cloud formed due to a sneeze, the Stokes number of the droplet is of the order St ∼ 1. In the present experimental study, the corn starch powder with a mean particle diameter of 14 *µ*m and a density of 500 kg/m^3^ is selected as a tracer particle. The Stokes number for the tracer particle at Re ∼ 30 000 is ∼1. The settling speed of the tracer particles estimated from the Stokes law is 3 mm/s. Hence, a 14 *µ*m particle travels 0.5 m in 166 s, which is close to the settling time reported by Verma *et al.* (2020a; 2020b) for fog as a tracer particle. We did not observe the settling of the particles due to gravity within this time. In a recent study by Verma *et al.* (2020a; 2020b), a flexible bellow filled with fog is pumped manually to simulate coughing at atmospheric pressure conditions. However, for sneezing, precise timing control of the order of 0.1 s is required. Such a high Reynolds number flow within 0.1 s is challenging to be generated at atmospheric pressure. Compressed air at high pressure in conjunction with valve timing control can generate such a high Reynolds number flow. However, it is challenging to compress fog at a higher pressure and mix appropriate quantities with dry compressed air to enable sufficient light scattering. Hence, we have used a solid tracer particle and ensured that the Stokes number is the same as that of the droplets. Since we achieved a dynamic similarity by matching Re, duration of the sneeze, and Stokes number, between the actual sneeze and our experiment, the estimation of travel of these tracer particles will be a reasonable representation of the actual sneeze scenario.

The solenoid valve’s opening timing is adjusted, and pressurized air supplied by the compressed air tank carries a sufficient amount of tracer particles to the orifice exit. A sheet of light formed by the laser source cuts the jet in the axial direction. A standard video camera (Canon EOS 6D DSLR) facing the laser sheet generated using a 5 mW laser captures the light scattered by tracer particles, and it is used to track the evolution of the sneeze.

The efficacy of several types of materials that are commonly used to cover faces such as N-95 masks, homemade cotton masks, and surgical masks is tested under the act of a sneeze. These protective measures are widely used by the majority of the population to control the spread of COVID-19, along with social distancing and frequent hand washing (Cheng *et al.*, 2020; Clase *et al.*, 2020; Wilder-Smith and Freedman, 2020; and Dbouk and Drikakis, 2020a; 2020b). Hence, these protectives measures’ efficacy is evaluated under an act of sneeze in the present work.

## RESULTS AND DISCUSSION

**FIG. 3.**
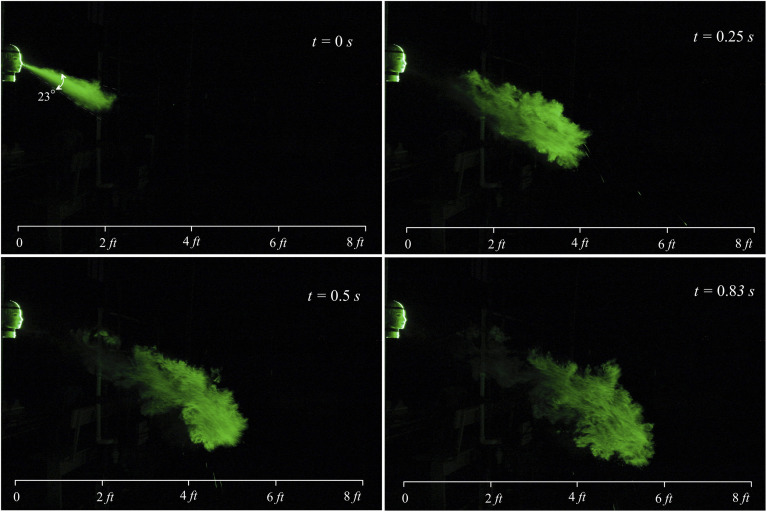
Evolution of a sneeze at Re = 30 000.

**FIG. 4.**
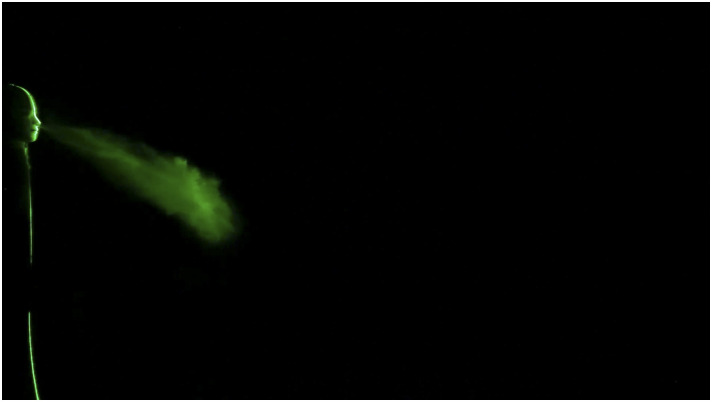
Image captured during evolution of the sneeze at Re = 30 000. Multimedia view: https://doi.org/10.1063/5.0030101.110.1063/5.0030101.1

In the present investigation, the evolution of sneezing and the effectiveness of different face masks and shields are analyzed through an experiment using a standard mannequin. The experimentation is carried out in a stagnant environment. The camera is positioned in both an inclined plane and a front plane to precisely capture the sneeze’s reach and evolution. The evolution of a typical sneeze is shown in Fig. 3 and Fig. 4 (Multimedia view). The dynamics of the turbulent jet is precisely captured in our visualization study. A sneeze resembles a free turbulent jet based on the shape and jet spreading angle. Like a turbulent jet, the shape of expelled particles is conical, and the spreading angle is ∼23°. The turbulent nature of the jet is visible immediately after the nozzle exit. The jet entrainment increases continuously with the downstream distance, and in just 0.5 s, the jet reaches 4 ft. This entrainment mechanism may impart moisture and heat, preventing the smaller droplets’ evaporation, which may travel a considerable distance as a tracer in a turbulent cloud (Bourouiba, 2020). The trajectory of the turbulent jet is inclined due to the inclination angle of the nose. The jet impinges on the floor at around 10 ft, reflects, and travels further in the streamwise direction. The velocity of the jet significantly reduces after the interaction with the floor.

**FIG. 5.**
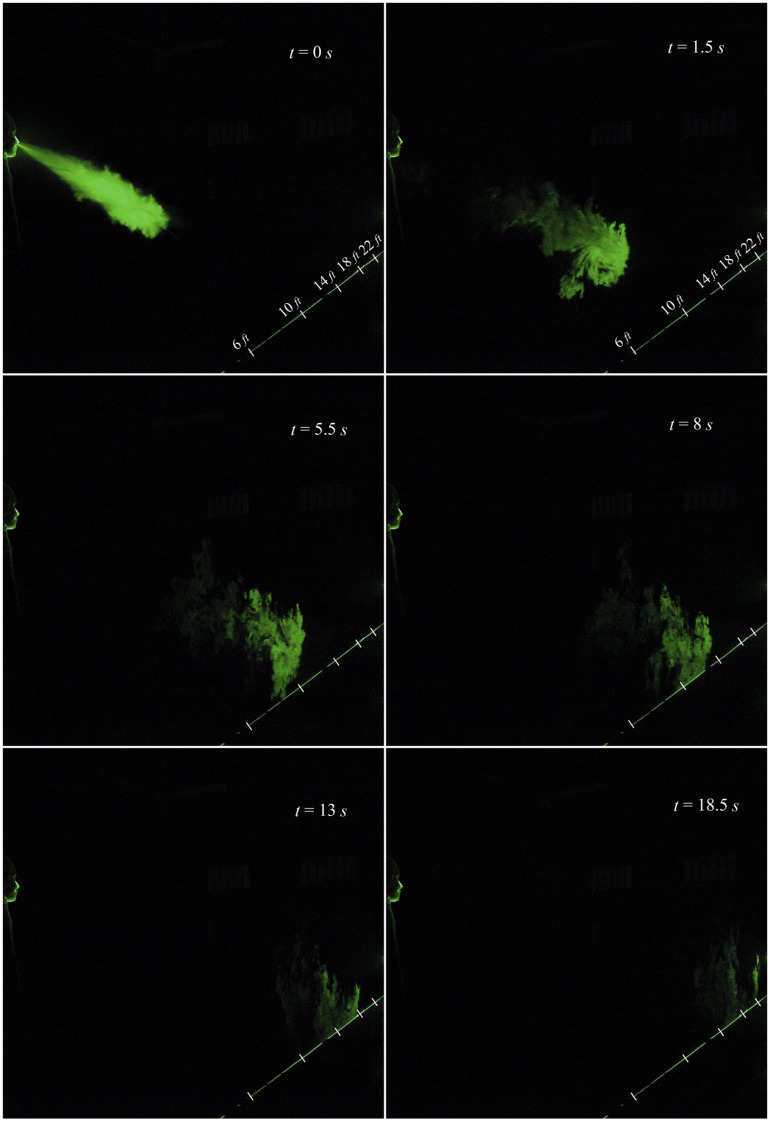
Reach of the sneeze at Re = 30 000 captured from the backside of the mannequin.

**FIG. 6.**
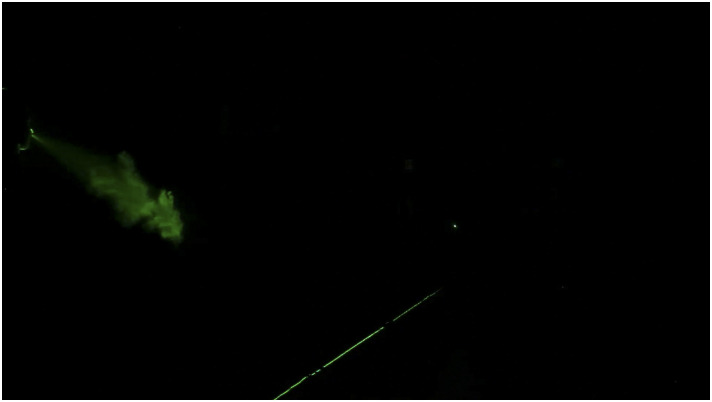
Image captured from an angle showing the reach of sneeze at Re = 30 000. Multimedia view: https://doi.org/10.1063/5.0030101.210.1063/5.0030101.2

We can infer that larger-sized droplets will fall on the floor before 10 ft (Das *et al.*, 2020). However, the smaller droplets will travel a considerable distance as freely suspended tracers in the turbulent cloud. The real droplets may not encounter a significant reflection. However, a small fraction of smaller diameter droplets may still get reflected and follow the turbulent cloud. The reach of the sneeze is shown in Fig. 5 and Fig. 6 (Multimedia view). It is interesting to observe that the jet’s reach is nearly 22 ft in 18.5 s, and in 22 s, the tracer particles are visible up to 25 ft; beyond this, the tracer particles get settled down on the floor or leave the visualization plane. Our results are in close agreement with the reach of the sneeze (23 ft–26 ft) reported by Bourouiba (2020). At 25 ft, we expect that only smaller diameter particles <14 *µ*m remain suspended in the turbulent cloud.

Recently, Pendar and Páscoa (2020) reported a social distancing of ∼13 ft based on a computational study for sneezing. However, their safe distance guideline is based on larger diameter droplets (∼100 *µ*m). Our experimental results clearly show the presence of particles at 25 ft. At this distance, the particles are near the ground, and hence, chances of transmission of infection are less. Any adverse environmental effects such as breeze or circulation caused by ventilation may transmit these particles to a healthy host. Hence, the present findings need further attention to reformulate the social distancing guidelines.

**FIG. 7.**
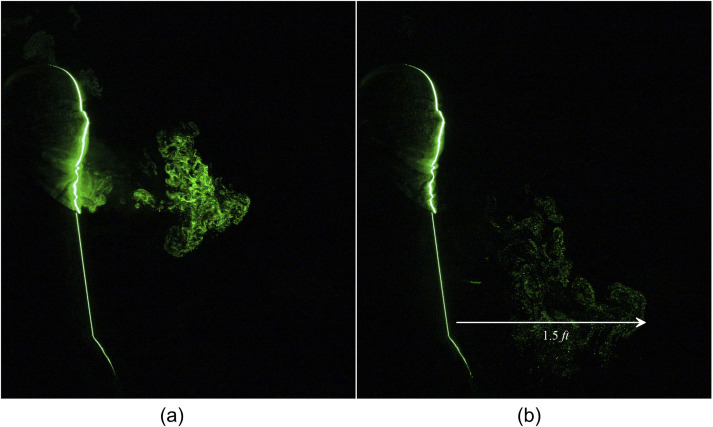
Leakage of a human sneeze from a two-layered triangle mask can travel to 1.5 ft. (a) t = 0.7 s and (b) t = 1.95 s after the emanation of sneeze.

**FIG. 8.**
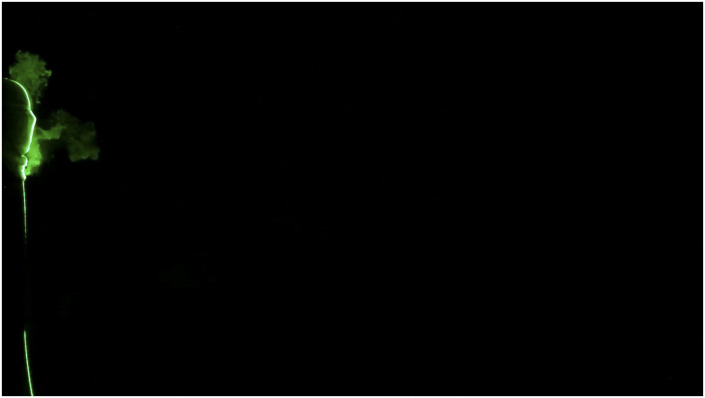
Leakage of a human sneeze from a two-layered triangle mask can travel to 1.5 ft. Multimedia view: https://doi.org/10.1063/5.0030101.310.1063/5.0030101.3

**FIG. 9.**
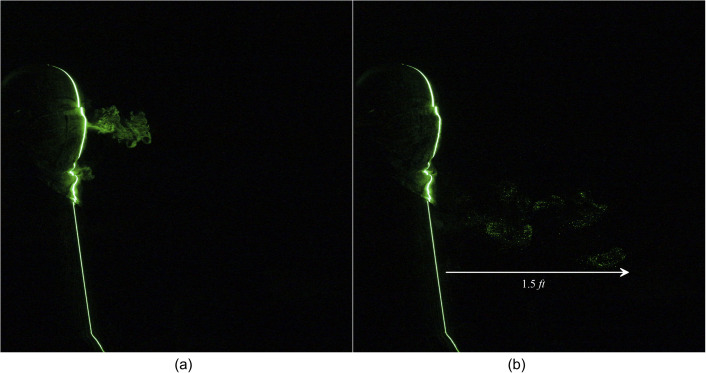
Considerable escape of human sneeze from a two-layered triangle mask with an additional layer of cotton stitched over it. The particles travel up to 1.5 ft. (a) t = 0 s and (b) t = 3.1 s after the emanation of sneeze.

**FIG. 10.**
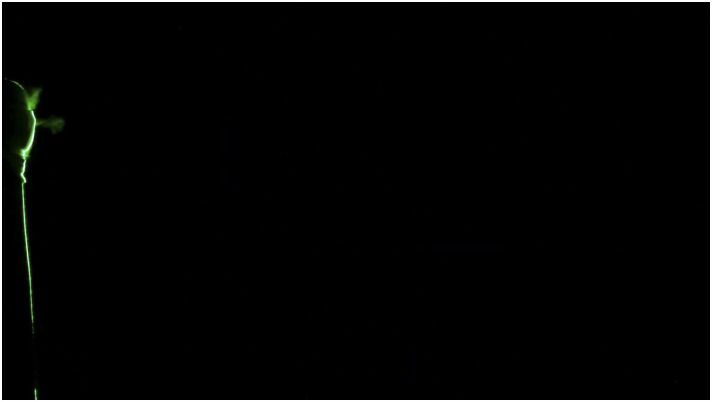
Leakage of a human sneeze from a three-layered triangle mask. The particles travel up to 1.5 ft. Multimedia view: https://doi.org/10.1063/5.0030101.410.1063/5.0030101.4

In most of the developing countries, the majority of the population is using non-standard masks, homemade masks, and handkerchiefs as a preventive measure to combat the spread of COVID-19. The efficacy of various standard and non-standard masks is also evaluated in our experiments. The spreading of human sneeze leaked from a two-layered triangle mask constructed using a handkerchief is shown in Fig. 7 and Fig. 8 (Multimedia view). From Fig. 7(b), it is observed that the emanated particles travel a distance of 1.5 ft in 1.95 s. The homemade triangle mask constructed by using a handkerchief significantly impedes the penetration of the particles; however, a noticeable leakage is observed in the forward direction. As expected, along the forward movement, the concentration of the tracer particles reduces significantly. The efficiency of two-layered triangle masks is further improved with an additional layer of cotton stitched over the triangle mask. It is observed that although the addition of extra material did not affect the distance traveled by the tracer particles, it could arrest a significant amount of tracer droplets, as shown in Fig. 9 and Fig. 10 (Multimedia view).

**FIG. 11.**
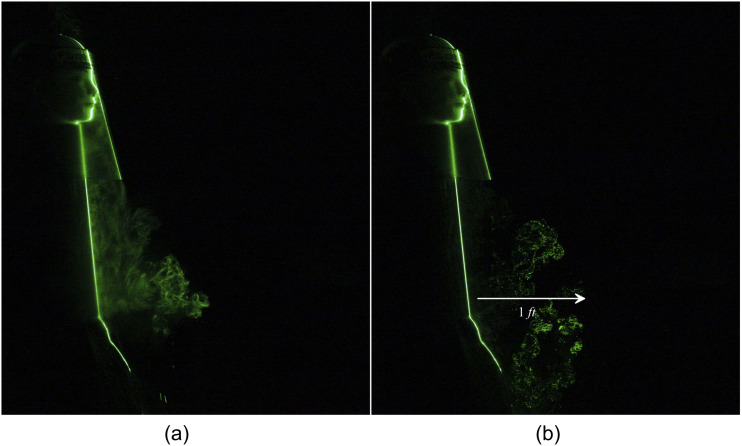
Escaped particles from a plastic face shield can travel to 1 ft. (a) t = 0 s and (b) t = 0.6 s after the emanation of sneeze.

**FIG. 12.**
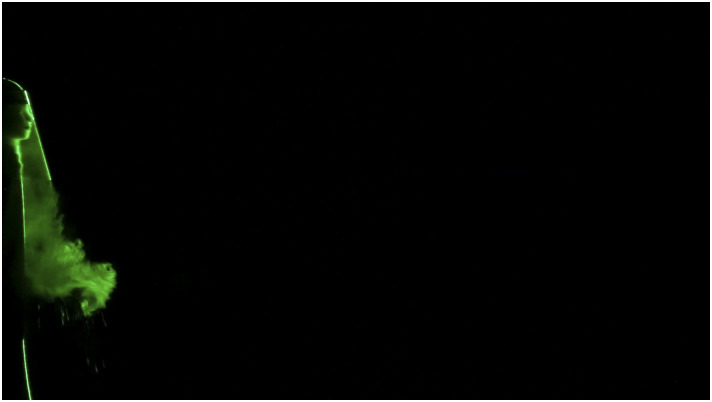
Escaped particles from a plastic face shield can travel to 1 ft. Multimedia view: https://doi.org/10.1063/5.0030101.510.1063/5.0030101.5

**FIG. 13.**
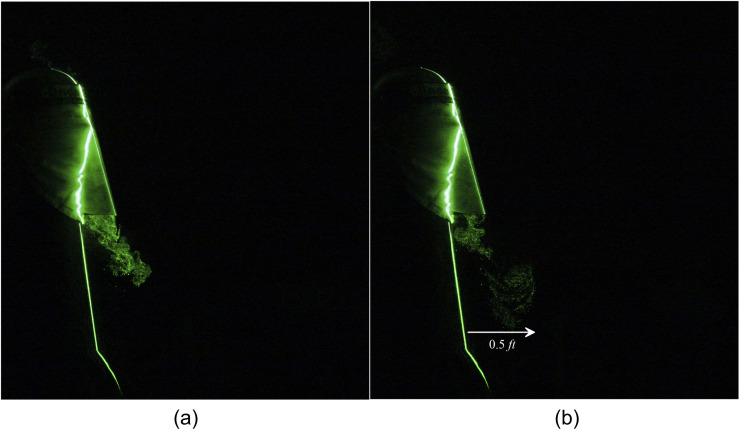
Downward leakage of a human sneeze from a two-layered triangle mask with a face shield can travel to 0.5 ft. Images taken at (a) t = 0.4 s and (b) t = 1.9 s after the emanation of sneeze.

**FIG. 14.**
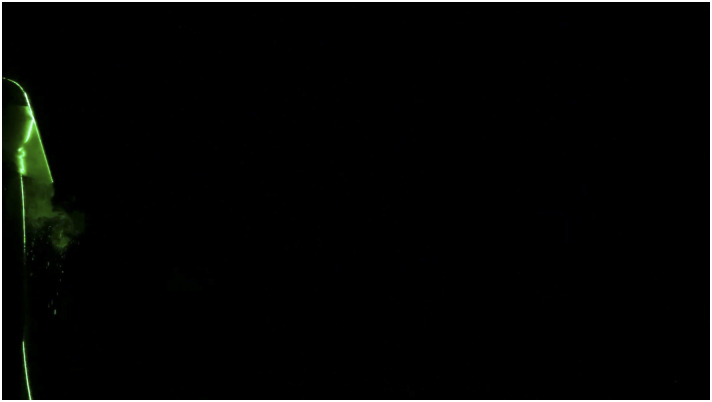
Downward leakage of a human sneeze from a two-layered triangle mask with a face shield can travel to 0.5 ft. Multimedia view: https://doi.org/10.1063/5.0030101.610.1063/5.0030101.6

A face shield is doing an excellent job of blocking the particles moving in the forward direction. However, Fig. 11(b) shows that a massive amount of particles are escaping below the face shield and travel a distance of 1 ft [Fig. 12 (Multimedia view)]. Hence, the face shield alone is not recommended for protecting the spreading of the virus. A face shield in conjunction with the two-layered triangle mask effectively restricts the leakage in the forward direction. This arrangement could entirely obstruct the forward movement of the jet shown in Fig. 13(a) and Fig. 14 (Multimedia view). Still, a significant loss of particles is noticed in the downward direction, which travel a noticeable distance of 0.5 ft [Fig. 13(b)].

**FIG. 15.**
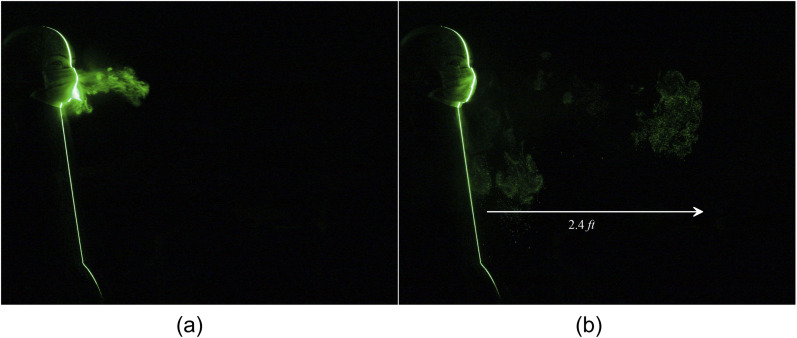
Leakage of tracer particles from a surgical mask. (a) t = 0 s and (b) t = 1.35 s after the emanation of sneeze.

**FIG. 16.**
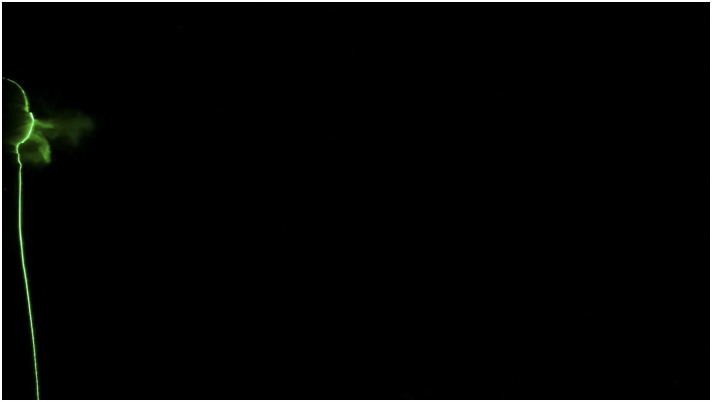
Leakage of tracer particles from a surgical mask. Multimedia view: https://doi.org/10.1063/5.0030101.710.1063/5.0030101.7

**FIG. 17.**
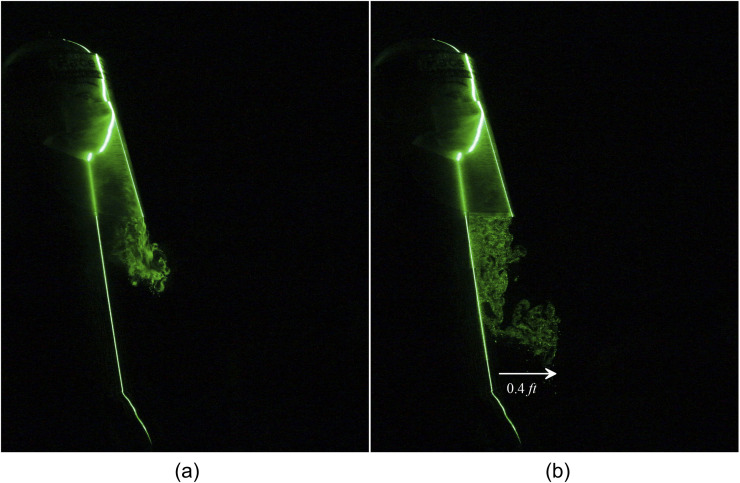
Leakage of tracer particles from a surgical mask with a face shield at (a) t = 0 s and (b) t = 0.45 s after the emanation of a sneeze.

**FIG. 18.**
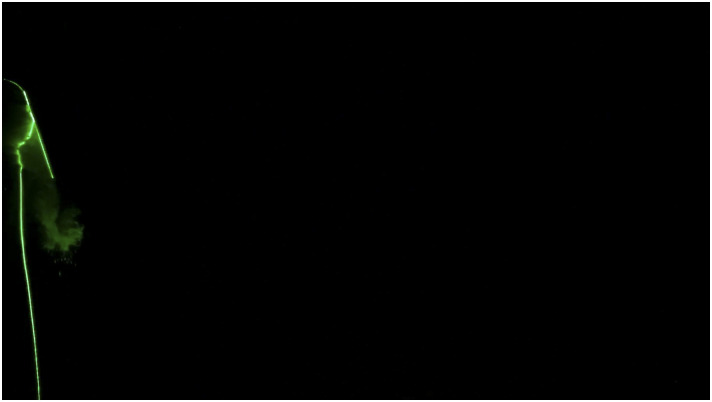
Leakage of tracer particles from a surgical mask with a face shield. Multimedia view: https://doi.org/10.1063/5.0030101.810.1063/5.0030101.8

A standard three-layer surgical mask seems to be the least effective means of preventing particle leakage. The leaked particles from the sneeze travel a distance of 2.5 ft, as shown in Fig. 15 and Fig. 16 (Multimedia view). A standard three-layer surgical mask with a face shield combination restricts the particles’ forward motion significantly; however, the particles leak in the downward direction up to 0.5 ft, as shown in Fig. 17 and Fig. 18 (Multimedia view). Such droplets will settle on the floor or nearby objects such as tables and chairs. Hence, it is mandatory to sanitize the tables, chairs, floor, etc., in offices, hospitals, and other public places more frequently even when people are wearing protective equipment such as face masks and shields. Our study indicates that non-standard two-layer and three-layer triangle masks constructed using a handkerchief are better than a standard three-layer surgical mask. However, complete prevention of the forward movement of the particles requires an additional face shield along with these, which may not be feasible for the general population to adopt in their daily routine.

Hence, we strongly recommend using the elbow or hands to prevent droplet leakage even after wearing a mask during sneezing and coughing.

**FIG. 19.**
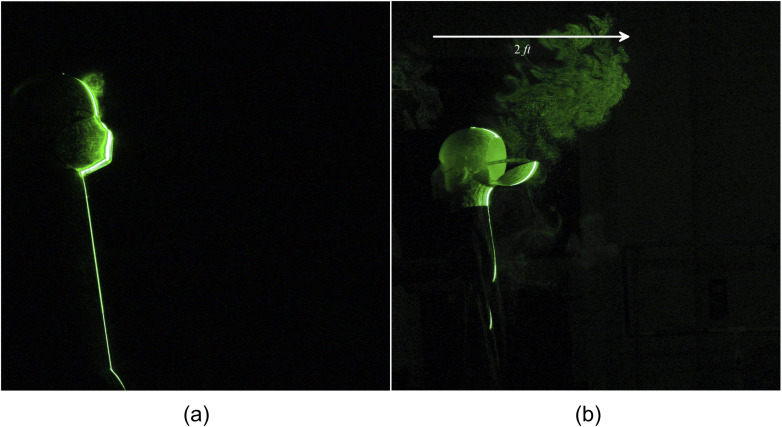
Leakage of human sneeze in the forward direction from an N-95 mask. Significant leakage in the backside and upward direction, which travels up to 2 ft. (a) t = 0 s and (b) t = 3.58 s after the emanation of sneeze. (a) Forward. (b) Backward.

**FIG. 20.**
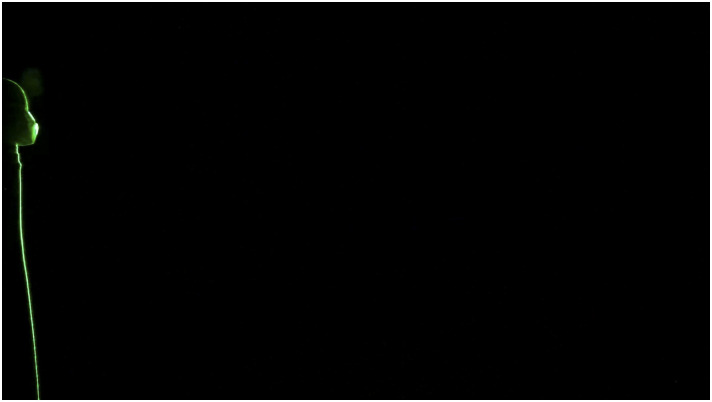
Leakage of tracer particles from an N-95 mask. Multimedia view: https://doi.org/10.1063/5.0030101.910.1063/5.0030101.9

Furthermore, an analysis of the most appreciated and adopted N-95 masks is also carried out. It is interesting to observe that an N-95 mask completely impedes the tracer particle leakage in the forward direction, as shown in Fig. 19(a) and Fig. 20 (Multimedia view). Surprisingly, it is observed that a significant amount of particles escaped from the gap between the nose and the mask. These leaked particles travel in the backward direction up to a distance of 2 ft in Fig. 19(b).

The estimated range strongly depends on the leakage rate from the top and sides, which may vary from person to person based on the mask’s fitment. These droplets can easily be sucked inside the heating, ventilation, and air conditioning (HVAC) ducts. We would like to emphasize that it is practically impossible to design a mask without leakage. Such a design may have severe implications on human health because it is not advisable to bear the internal impact on organs due to the sneeze’s complete blockage. However, a proper design may significantly reduce the leakage from the top and sides. Hence, it is strongly advised to follow social distance from all orientations. Moreover, it is recommended to evacuate the location immediately where an act of sneeze occurred. The summary of various results is provided in Table 1.

**TABLE I.** Summary of different masks, types of materials used, number of layers or threads/inch present, and average distance traveled by the tracer particles beyond which their presence is unnoticeable.

Type of mask	Material	Number of layers or threads/in.	Average distance traveled by sneeze
Without mask	…	…	∼25 ft
Two-layered mask	Cotton	50 threads/in.	∼1.5 ft
Three-layered mask	Cotton	65 threads/in.	∼1.5 ft
Face shield	Polycarbonate	…	∼1 ft
Two-layered mask with a face shield	…	50 threads/in.	∼0.5 ft
Surgical mask	Polypropylene	Three layered	∼2.5 ft
Surgical mask with a face shield	…	…	∼0.4 ft
N-95	Synthetic polymer fibers	Five layered	0 ft in the forward direction
			∼2 ft in the backward direction

The results reported in this study are in a quiescent environment. However, the droplets’ reach may further increase with circulation caused by ventilation in closed rooms and breeze in open areas. Hence, we recommend a social distancing of at least 6 ft along with protective measures such as face masks and face shields to minimize the spread of droplets carrying infectious pathogens responsible for COVID-19 and other similar diseases.

In conclusion, we have demonstrated a simple experimental setup to simulate sneeze. In our experiments, we have ensured dynamic similarity by matching Re, duration of the sneeze, and Stokes number, between the actual sneeze and our experiment. The estimation of travel of these tracer particles is a reasonable representation of the actual sneeze scenario. A typical sneeze closely resembles a turbulent jet and can travel up to 25 ft in nearly 22 s. The present widely accepted safe distance of 6 ft is highly underestimated, especially under the act of a sneeze. Like a turbulent jet, the shape of expelled particles is conical, and the spreading angle is ∼23°.

None of the widely adopted projective measures such as homemade two-layer and three-layer masks, standard three-layer surgical masks, and face shields effectively block the escape of particles ejected during sneezing. However, these projective measures effectively reduce the leakage and reach of the sneeze within 1 ft–3 ft. It is interesting to note that an N-95 mask completely impedes the forward leakage of the particles. However, leakage from the sides is inevitable, and the leaked particles can travel up to 2 ft in the backward direction.

These droplets can easily be sucked inside the heating, ventilation, and air conditioning (HVAC) ducts. It is practically impossible to design a mask without leakage. Such a design may have severe implications on human health because it is not advisable to bear the internal impact on organs due to the sneeze’s complete blockage. However, a proper design may significantly reduce the leakage from the top and sides. Hence, it is strongly advised to wear protective measures such as face masks and shields and to follow a social distance of at least 6 ft from all orientations. We strongly recommend using the elbow or hands to prevent droplets’ leakage even after wearing a mask during sneezing and coughing. Moreover, it is recommended to evacuate the location immediately where an act of sneeze occurred.

## Data Availability

The data that support the findings of this study are available from the corresponding author upon reasonable request.
